# The association between physical activity and sexual dysfunction in patients with diabetes mellitus of European and South Asian origin: The Oxford Sexual Dysfunction Study

**DOI:** 10.1186/s40001-015-0186-5

**Published:** 2015-11-05

**Authors:** Lasantha S. Malavige, Pabasi Wijesekara, Priyanga Ranasinghe, Jonathan C. Levy

**Affiliations:** Nuffield Department of Clinical Medicine, University of Oxford, Oxford, OX3 7LJ UK; Oxford Radcliffe Trust, Oxford Centre for Diabetes, Endocrinology and Metabolism, Oxford, UK; Ministry of Health Care and Nutrition, Colombo, Sri Lanka; Department of Pharmacology, Faculty of Medicine, University of Colombo, Colombo, Sri Lanka

**Keywords:** Physical activity, Diabetes, Erectile dysfunction, South Asian, Europid

## Abstract

**Background:**

The present study aims to evaluate the relationship between physical activity and sexual dysfunction amongst an ethnic South Asian population living in the United Kingdom and compare the association with that of the native Caucasian population.

**Methods:**

Twenty-five general practitioner clinics from eight primary care trusts in the United Kingdom collaborated in the Oxford Sexual Dysfunction Study. In each practice, a sample of diabetic and non-diabetic patients of European/Europid and South Asian origin were invited for the study. Erectile dysfunction (ED) was assessed using a five-item version of the International Index of Erectile Function. Premature ejaculation (PE) was diagnosed using the premature ejaculation diagnostic tool. Libido was assessed by asking participants to grade their desire for sexual activity. Physical activity during the past week was assessed using the short version of the International Physical Activity Questionnaire (IPAQ). A binary logistic regression analysis was performed in all adults, Europids and South Asians with ‘presence of ED’ as the dichotomous dependent variable (0 = ED absent; 1 = ED present) and age, diabetes status, physical activity, ethnicity, current smoking and use of antihypertensive medications as the independent variables.

**Results:**

Sample size was 510, and mean age was 56.9 ± 9.7 years. There were 63.9 % (*n* = 326) Europid males in the study population. The prevalence of ED was 64.5 % and it was significantly higher in men with diabetes than in those without diabetes (84.4 vs. 49.0 %, *p* < 0.001). The overall prevalence of PE was 28.8 %, (with diabetes 32.6 %, without diabetes 25.8 %; *p* = 0.109). Reduced libido was reported by 26.9 % of study participants (with diabetes 32.8 %, without diabetes 22.0 %; *p* < 0.01). The median (IQR) total physical activity of the study population was 2373 (3612) MET-min/week. In the IPAQ categorical score, 36.8 % (*n* = 184/434) males were ‘highly active’, and 17.8 % (*n* = 89/434) were ‘inactive’. In all adults, age (OR: 1.06), South Asian ethnicity (OR: 1.40), physical inactivity (OR: 1.62) and presence of diabetes (OR: 3.90) all were associated with significantly increased risk of developing ED. A similar result was observed in Europids but not in South Asians.

**Conclusions:**

Erectile dysfunction was associated with physical inactivity, mainly in Europid males, irrespective of diabetes status. This association was not observed in South Asian males with or without diabetes.

## Background

Diabetes mellitus is a chronic non-communicable disease associated with a host of micro- and macro-vascular complications that increases morbidity and mortality, while also compromising quality of life [1]. Sexual dysfunction is commonly seen in patients with diabetes mellitus. The prevalence of erectile dysfunction (ED) amongst diabetic men is estimated to be around 35–90 % [2]. However, in contrast to men without diabetes, ED among patients with diabetes tends to occur 10–15 years earlier, is more severe, associated with a poorer quality of life, and is less responsive to treatment [3–5]. This probably is a result of the multi-factorial aetio-pathogenesis of ED in patients with diabetes, where vascular, neurological and endocrine abnormalities act synergistically [6]. Hence, sexual dysfunction amongst patients with diabetes constitutes a complex and differential entity in forms of aetio-pathogenesis, progression and treatment. Sexual dysfunctions associated with diabetes are also known to compromise quality of life in both males and females [7, 8]. In addition, ED is also known to precede cardiovascular clinical events in males [9]. Therefore, it is important to study these sexual dysfunctions in diabetes and their associations in order to identify differential risk factors and formulate evidence-based management guidelines.

The association between diabetes mellitus and physical activity is well documented and strongly supported by research evidence. Increased levels of physical activity are known to reduce the risk of diabetes [10]. In addition, studies have also shown that an active and fit way of life substantially delays the progression from a state of impaired glycaemic control to frank diabetes [11]. There is compelling observational evidence that higher levels of physical activity and cardio-respiratory fitness confers a substantial protection against mortality and premature cardiovascular disease in individuals with diabetes [12]. Sexual dysfunction in diabetes is also known to be associated with physical activity. Leisure time and work-related physical activity has shown a protective effect on ED among men with diabetes [13]. Higher levels of physical activity have also shown a protective effect against female sexual dysfunction associated with diabetes [14].

Several studies have highlighted an ethnic disparity in the susceptibility towards diabetes and its complications, for example South Asians are known to have an increased predisposition for type II diabetes mellitus [15]. In UK, the risk of diabetes is five times higher for immigrants from Pakistan and Bangladesh and three times higher for Indian immigrants, with an associated increased risk of complications, morbidity and mortality compared with the native white Caucasian population [16]. Progression of diabetes is also known to be more rapid among South Asians, the decline in glycaemic control over time was much more rapid among South Asians when compared to Europeans [17]. In addition to the large populations in South Asia which comprises one-fourth of the worlds’ population, a significant number of immigrants from the region are living in affluent Western nations [18]. As a consequence, a disease such as type II diabetes mellitus affecting the ethnic South Asian sub-population will have potential implications on global health. Therefore, it is important to identify differential risk factors affecting disease susceptibility and progression among South Asians. Although there are studies exploring the associations between sexual dysfunction, diabetes and physical activity among Caucasians, at present there are no studies on an ethnic South Asian population [13, 14]. The present study aims to evaluate the relationship between physical activity and sexual dysfunction amongst an ethnic South Asians population living in the United Kingdom and compare the association with that of the native Caucasian population.

## Methods

### Study population and sampling

Detailed sampling has been described elsewhere, in brief 25 general practitioner (GP) clinics from eight primary care trusts (PCTs) in the United Kingdom collaborated in the Oxford Sexual Dysfunction Study [19]. In each practice, a sample of diabetic and non-diabetic patients of European and South Asian origin were selected for invitation to participate in the study. The term “Europid” is used to denote people of European origin. This primary sample was sent invitation letters, the patient information sheet, and consent forms, with a request that the consent form be filled in and returned to the practice. Consenting individuals from the primary sample were then sent the study questionnaires. Subjects were excluded from analysis if they had previous prostate surgery, pelvic irradiation, or spinal cord injury or if their ethnic origin was other than SA or Europid. Ethical approval for this study was obtained from Oxfordshire Research Ethics Committee C. Research and Development approval was obtained from all participating PCTs.

### Data collection and cleaning

All scales used in the study questionnaires were translated and linguistically validated into Punjabi, Urdu, Hindi, Tamil, and Sinhala languages. ED was assessed using a five-item version of the International Index of Erectile Function (IIEF-5), also known as the Sexual Health Inventory for Men [20]. ED was categorized into five grades of severity on the basis of the IIEF-5 score, 22–25 (normal erectile function), 17–21 (mild ED), 12–16 (mild to moderate ED), 8–11 (moderate ED), and 1–7 (severe to complete ED). For analytical purposes, a binary variable was also created where an IIEF-5 score of 22–25 was considered normal and 1–21 was considered as having ED. PE was diagnosed using the premature ejaculation diagnostic tool (PEDT) [21]. The scores were then categorized into “no PE” (PEDT score 0–8), “probable PE” (PEDT score 9–10), and “PE” (PEDT score 11–20). In addition, a binary variable was created for the purpose of analysis, where a PEDT score of 0–10 was taken as not having PE and scores of 11 and above taken as having PE. Libido was assessed by asking participants to grade their desire for sexual activity as either “very high”, “high”, “moderate”, “low” or “very low/none”. These five categories were subsequently combined as normal libido (“moderate”, “high”, or “very high”) and reduced libido (“low” or “very low/none”) for the purpose of analysis.

Physical activity during the past week was assessed using the short version of the International Physical Activity Questionnaire (IPAQ) [22]. The short IPAQ allows categorical and continuous measurements of physical activity. The continuous score allows the estimation of the weekly energy expenditure expressed in MET-min/week (metabolic equivalent-minutes). The categorical score classifies individual into three categories; ‘inactive’, ‘moderately active’ and ‘highly active’. Records maintained by the GP practice were used to ascertain anthropometric, clinical, and biochemical data.

Data cleaning was done in accordance with the IPAQ data processing guideline [23]. The data processing guideline of the IPAQ is used to exclude outlier data, recode minimum values and deal with high values. These guidelines ensure that highly active people remain classified as ‘highly active’, while decreasing the chances that less active individuals are misclassified and coded as ‘highly active’. Furthermore, given the non-normal distribution of energy expenditure in many populations, the continuous indicator (MET-min/week) is presented as median values with inter-quartile range (IQR) rather than means, as recommended by the IPAQ data processing guideline [23].

### Data analysis

Data were analyzed using SPSS version 17.0 (SPSS Inc., Chicago, IL, USA). The significance of the differences between proportions and means was tested using *z* test and Student’s *t* test or ANOVA, respectively. Subjects were divided into two groups based on IPAQ categorical score, the ‘moderately active’ and ‘highly active’ groups were combined to one group (‘physically active’) and the ‘inactive’ group remained the same (‘physically inactive’). A binary logistic regression analysis was performed in all adults with ‘presence of ED’ as the dichotomous dependent variable (0 = ED absent; 1 = ED present) and age, diabetes status (0 = absent, 1 = present), physical activity (0 = physically inactive, 1 = physically active), ethnicity (0 = Europid, 1 = South Asian), current smoking (0 = non-smoker, 1 = smoker) and use of antihypertensive medications (0 = no, 1 = yes) as the independent variables. The explanatory independent variables that were associated with the dependent variable in univariate analysis (*p* < 0.25) were selected to be included in the regression analysis. The explanatory variables selected above were subsequently included in a binary logistic regression model, a backward elimination procedure was used and a *p* value of 0.10 was considered as the cut-off for removal of variables. A similar binary logistic regression analysis with above dependant and independent variables was also performed separately for both Europids and South Asians separately. In all statistical analyses, a *P* < 0.05 was considered significant.

## Results

### Sample characteristics

Sample size was 510, and mean age was 56.9 ± 9.7 years (range 20–72). There were 63.9 % (*n* = 326) Europid males in the study population. There was no significant difference in mean age between Europids (56.5 ± 8.9 years) and South Asians (56.9 ± 10.9 years). The prevalence of diabetes, hypertension and ischaemic heart disease in the population were 45.5 % (*n* = 232/510), 53.2 % (*n* = 260/489) and 16.2 % (*n* = 79/489), respectively. The prevalence of hypertension and ischaemic heart disease were both higher among men with diabetes than those without (*p* < 0.05); however, there was no significant ethnic variation between Europids and South Asians [19]. The overall prevalence of smoking was 54.9 % (*n* = 280), and it was significantly higher in Europid males (66.5 %) than South Asians (34.8 %) (*p* < 0.05). This was observed in both males with diabetes (69.5 vs. 37.5 %) and without diabetes (64.1 vs. 31.3 %). Further details of the baseline characteristics are summarized elsewhere [19].

### Prevalence of sexual dysfunction

The prevalence of ED as assessed by the IIEF (score 1–21) was 64.5 % and it was significantly higher in men with diabetes than in those without diabetes (84.4 vs. 49.0 %, *p* < 0.001). In men with diabetes the prevalence of ED was similar in Europids and South Asians (84.1 vs. 84.8 %); however, in men without diabetes, South Asians had a higher prevalence of ED (62.3 vs. 44.1 %) (*p* < 0.05). The overall prevalence of PE was 28.8 %, (with diabetes 32.6 %, without diabetes 25.8 %; *p* = 0.109). More South Asian men reported PE (with diabetes 45.8 %, without diabetes 41.9 %) than their Europid counterparts (with diabetes 22.4 %, without diabetes 20.2 %), irrespective of diabetes status (*p* < 0.001). Reduced libido was reported by 26.9 % of study participants (with diabetes 32.8 %, without diabetes 22.0 %; *p* < 0.01). However, there was no significant difference in reduced libido between the ethnic groups.

### Physical activity of the study population

The median (IQR) total physical activity of the study population was 2373 (3612) MET-min/week. In the IPAQ categorical score, 36.8 % (*n* = 184/434) males were ‘highly active’, and 17.8 % (*n* = 89/434) were ‘inactive’. Europid males had a median (IQR) total physical activity level of 2680.5 (3974.63) MET-min/week, which was significantly higher than the total physical activity level of South Asians [1680 (3271) MET-min/week] (*p* < 0.05). However, this difference was not observed independently in patients with and without diabetes. Among Europids 45.7 % were ‘highly active’, compared to 36.4 % among South Asians (*p* < 0.05) in the IPAQ categorical score. This difference was not significant in the ‘moderately active’ and ‘inactive’ groups. There was no significant difference in the IPAQ continuous and categorical scores in males with and without diabetes.

### Physical activity, sexual dysfunction and diabetes

In all males median MET-min/week was significantly lower in those with ED (*p* < 0.01) (Table 1). A similar pattern was observed in Europids when considered separately, but not in South Asians. In patients with diabetes, a lower median MET-min/week was observed in those with ED (*p* < 0.05) (Table 1). In Europid males with diabetes, those with ED had a lower median MET-min/week; however, this was not observed in South Asians. A similar relationship was not observed for ED in patients without diabetes. In all males and in Europids a significantly lower percentage of those with ED were in the highly active group in the IPAQ categorical score (Table 2). This was not observed in South Asians.

**Table 1:** Median physical activity level and sexual dysfunction in all males, Europids and South Asians

	Median MET-min/week (IQR)								
	Erectile dysfunction		*p* value	Premature ejaculation		*p* value	Libido		*p* value
	Absent	Present		Absent	Present		Normal	Reduced	
All males									
All	2772 (4122)	2066 (3532)	<0.01	2400 (3851)	2079 (3465)	0.226	2373 (3573)	2400 (3921)	0.916
Europids	3355 (4340)	2493 (3768)	<0.01	2763 (4382)	2652 (3400)	0.283	2706 (4076)	2641 (3846)	0.929
South Asians	1985 (2841)	1662 (3142)	0.184	1782 (3442)	1638 (3465)	0.931	1710 (2925)	1584 (3726)	0.909
Patients with diabetes									
All	3816 (7492)	1980 (3472)	<0.05	2424 (3711)	2079 (4358)	0.307	2163 (3721)	2148 (3465)	0.887
Europids	6075 (7539)	2446 (3478)	<0.01	2739 (3336)	2706 (5095)	0.168	2772 (5013)	2400 (3057)	0.652
South Asians	1638 (2940)	1674 (3560)	0.694	1680 (3231)	1538 (4346)	0.974	1674 (3123)	1584 (4510)	0.910
Patients without diabetes									
All	2706 (3444)	2333 (4050)	0.109	2272 (4449)	2493 (2712)	0.686	2382 (3420)	2655 (4180)	0.644
Europids	2836 (4125)	2641 (4191)	0.271	2772 (5174)	2598 (2475)	0.827	2706 (3706)	3093 (3515)	0.401
South Asians	1853 (2772)	1538 (3025)	0.361	1737 (3699)	1809 (3304)	0.879	1746 (3063)	1280 (3722)	0.398

*IQR* inter-quartile range

**Table 2:** IPAQ categorical scores and sexual dysfunction in all males, Europids and South Asians

	Percentage (95 % CI)								
	Erectile dysfunction		*p* value	Premature ejaculation		*p* value	Libido		*p* value
	Absent	Present		Absent	Present		Normal	Reduced	
All males									
Highly active	53.7 (44.4–62.7)	37.9 (32.5–43.6)	<0.01	44.7 (39.1–50.4)	38.5 (29.1–48.5)	0.304	43.4 (37.9–48.9)	40.4 (31.1–50.2)	0.654
Moderately active	31.7 (23.6–40.7)	39.2 (33.8–44.9)	0.153	35.5 (30.2–41.0)	40.4 (30.9–50.5)	0.411	36.5 (31.3–42.0)	38.5 (29.4–48.3)	0.732
Inactive	14.6 (8.9–22.1)	22.8 (18.3–27.9)	0.065	19.8 (15.5–24.7)	21.2 (13.8–30.3)	0.779	20.1 (15.9–24.9)	21.1 (13.9–29.9)	0.891
Europids									
Highly active	57.3 (46.4–67.7)	40.2 (33.2–47.6)	<0.01	47.7 (40.9–54.6)	40.4 (27.0–54.9)	0.358	47.6 (40.7–54.6)	39.7 (28.0–52.3)	0.266
Moderately active	28.1 (19.1–38.6)	36.0 (29.1–43.3)	0.221	31.9 (25.8–38.6)	40.4 (27.0–54.9)	0.256	31.4 (25.2–38.2)	39.7 (28.0–52.3)	0.237
Inactive	14.6 (8.0–23.7)	23.8 (17.9–30.6)	0.084	20.4 (15.2–26.4)	19.2 (9.6–32.5)	0.924	21.0 (15.7–27.1)	20.6 (11.7–32.1)	0.932
South Asians									
Highly active	44.1 (27.2–62.1)	34.1 (26.1–43.6)	0.319	38.1 (28.5–48.6)	36.5 (23.6–51.0)	0.861	35.4 (26.6–44.9)	41.5 (26.3–57.9)	0.572
Moderately active	41.2 (24.6–59.3)	44.3 (35.3–53.6)	0.846	43.3 (33.3–53.8)	40.4 (27.0–54.9)	0.862	46.0 (36.6–55.6)	36.6 (22.1–53.1)	0.359
Inactive	14.7 (5.0–31.1)	21.3 (14.4–29.6)	0.473	18.6 (11.4–27.7)	23.1 (12.5–36.8)	0.526	18.6 (11.9–27.0)	22.0 (10.6–37.6)	0.650

In all males with diabetes 37.0 % of males with ED were highly active in comparison to 51.9 % males without ED who were highly active in the IPAQ categorical score (*p* < 0.01). Similarly in Europid males with diabetes and ED 35.6 % were highly active, as compared to 71.4 % of diabetic males without ED (*p* < 0.01). This relationship was not observed in South Asians and patients without diabetes. PE and libido did not show any distinct relationship with median MET-min/week (Table 1) and IPAQ categorical score (Table 2) in all adults, Europids and South Asians, irrespective of diabetes status.

### Results of the logistic regression analysis

The results of the binary logistic regression analysis in all adults using the dichotomous variable ‘presence of ED’ as the dependant factor and other independent variables are shown in Table 3. The overall model was statistically significant and the Cox and Snell *R*^2^ and Nagelkerke *R*^2^ values were 0.232 and 0.384, respectively. The results indicate that in all adults, age (OR: 1.06), South Asian ethnicity (OR: 1.40), physical inactivity (OR: 1.62) and presence of diabetes (OR: 3.90) all were associated with significantly increased risk of developing ED (Table 3). A similar result was observed in Europids. However, in South Asians physical inactivity was not associated with an increased risk of ED (Table 3).

**Table 3:** Binary logistic regression analysis of erectile dysfunction in all adults, Europids and South Asians

Co-variants	Odds ratio (95 % CI)		
	All adults	Europids	South Asians
South Asian ethnicity	1.40 (1.02–1.78)^#^		
Age	1.06 (1.04–1.09)^#^	1.05 (1.02–1.09)^#^	1.09 (1.01–1.19)^#^
Physically inactive	1.62 (1.22–2.02)*	1.89 (1.06–3.40)*	1.54 (0.28–8.62)
Presence of diabetes	3.90 (2.30–6.62)*	5.48 (2.76–10.90)*	6.09 (3.65–9.93)*
Current smoking	0.98 (0.97–1.00)	1.02 (0.99–1.04)	0.91 (0.74–1.07)
Use of antihypertensives	0.82 (0.56–1.21)	1.03 (0.84–1.22)	0.94 (0.15–5.98)

* *p* < 0.001; ^#^ *p* < 0.05

## Discussion

The results from the Oxford Sexual Dysfunction Study demonstrate that ED was associated with physical inactivity, mainly in Europid males. Europid males with ED had a lower median weekly MET-min in the IPAQ continuous score and were less ‘highly active’ in the IPAQ categorical score. Physical inactivity was associated with ED in Europid males in the binary logistic regression analysis, controlling for confounders such as age, diabetes, and use of antihypertensive medications. However, a similar association was not observed in South Asians males with ED. Other sexual dysfunctions such as PE and loss of libido did not demonstrate any significant relationship with physical activity, in both ethnicities, irrespective of diabetes status.

According to a recent systematic review on physical activity and ED conducted in 2015, both observational and interventional studies (including randomized controlled trials) from Austria, Australia, Brazil, China, Finland, Italy, Romania and USA have clearly demonstrated a relationship between physical activity and ED in males [24]. The odds ratio for physical inactivity/low physical activity observed in Australia (1.5) and China (1.13–1.67), were similar to that of the Europid males from UK (1.86) in the present study [24]. In addition, high levels of physical activity has demonstrated a protective effect, reducing the occurrence of ED in Australia (OR: 0.47), Austria (OR: 0.17), Finland (OR: 0.12), Italy (OR: 0.8) and USA (OR: 0.7) [24]. This review also showed that the intensity of physical activity is associated with ED levels, with the greatest benefits resulting from moderate to vigorous levels of physical activity [24]. Studies have shown that physical activity ameliorates ED by multiple mechanisms, including; improved cardiovascular fitness and endothelial dysfunction [25], increase in endothelial-derived NO [26], decrease in oxidative stress [27] and increase in regenerative endothelial progenitor cells (EPCs) [28].

Our results also show that the relationship between physical activity and ED is similar even for Europid males with diabetes. This has been observed earlier in males with metabolic syndrome and ED [29]. Previous studies have also shown that, in hypertensive patients with ED, an 8-week exercise training for the duration of 45–60 min per day improved ED compared with controls who remained sedentary [30]. It is well known that ED is a strong predictor of poor quality of life in males with type 2 diabetes mellitus [31, 32]. Furthermore, in males with type 2 diabetes without clinically overt cardiovascular disease, the presence of ED predicts a new onset of coronary heart disease (CHD) event [33, 34]. It is recommended that symptoms of ED be independently sought to identify high-risk subjects for comprehensive cardiovascular assessments [33]. Hence, physical activity interventions in males with diabetes and ED, will not only improve quality of life, but will also help in reducing diabetes-related complications and their associated morbidity and mortality.

We did not observe any significant relationship between ED/PE/reduced libido and physical activity in South Asian males, both with and without diabetes. We were also unable to find any previous studies in literature that evaluated the association between physical activity and ED in ethnic South Asian males with and without diabetes. This observation could have been due to the inaccuracies in the measurement of physical activity in South Asians using the IPAQ. Hall and co-workers demonstrated that South Asians compared to Europeans, exhibited significantly lower fat oxidation and the same level of fat oxidation at the same relative intensities of exercise, but higher carbohydrate oxidation at the same absolute exercise intensities after adjusting for age, BMI and fat mass [35]. Therefore, using similar MET values which were originally developed from Caucasians to estimate energy expenditure in South Asians may be erroneous. Furthermore, the coding of physical activity by type and intensity for South Asians is an area that has not been thoroughly studied at present. In addition South Asians are an ethnic group in which the willingness to discuss sensitive sexual issues is considered less than the Europids due to cultural reasons, even in postal survey as in the present study.

There are several limitations to our study, the cross-sectional design of our study can only demonstrates an association between ED and identified risk factors, and limits the inference of causality. Therefore, it is important to conduct prospective studies in healthy adults and in newly diagnosed young-onset adult patients with diabetes without ED and look for causality during subsequent follow-up [36]. Furthermore, it is well known that self reported measures can over-estimate physical activity, and the IPAQ may do this more than other physical activity questionnaires [37, 38]. Another limitation is that the MET values of some activities are not derived from actual oxygen consumption [39].

## Conclusions

The results from the Oxford Sexual Dysfunction Study demonstrate that erectile dysfunction was associated with physical inactivity, mainly in Europid males, irrespective of diabetes status. This association was not observed in South Asian males with or without diabetes. Other sexual dysfunctions such as premature ejaculation and loss of libido did not demonstrate any significant relationship with physical activity, in both ethnicities, irrespective of diabetes status.
